# iPSCs chondrogenic differentiation for personalized regenerative medicine: a literature review

**DOI:** 10.1186/s13287-024-03794-1

**Published:** 2024-06-26

**Authors:** Eltahir Abdelrazig Mohamed Ali, Rana Smaida, Morgane Meyer, Wenxin Ou, Zongjin Li, Zhongchao Han, Nadia Benkirane-Jessel, Jacques Eric Gottenberg, Guoqiang Hua

**Affiliations:** 1 Institut National de la Santé et de la Recherche Médicale (INSERM), UMR 1260, Regenerative NanoMedicine (RNM), 1 Rue Eugène Boeckel, 67000 Strasbourg, France; 2 Université de Strasbourg, 67000 Strasbourg, France; 3 Lamina Therapeutics, 1 Rue Eugène Boeckel, 67000 Strasbourg, France; 4 Nankai University School of Medicine, Tianjin, 300071 China; 5 Beijing Engineering Laboratory of Perinatal Stem Cells, Beijing Institute of Health and Stem Cells, Health & Biotech Co, Beijing, 100176 China; 6 Centre National de Référence des Maladies Auto-Immunes et Systémiques Rares, Est/Sud-Ouest (RESO), Service de Rhumatologie, Centre Hospitalier Universitaire de Strasbourg, 67000 Strasbourg, France; 7 Chongqing Medical University, 1 Yixueyuan Road, Yuzhong District, Chongqing, 400016 China

**Keywords:** Induced pluripotent stem cells, Chondrocytes, Mesenchymal stem cells, Osteoarthritis, Cartilage regeneration, Personalized regenerative medicine

## Abstract

Cartilage, an important connective tissue, provides structural support to other body tissues, and serves as a cushion against impacts throughout the body. Found at the end of the bones, cartilage decreases friction and averts bone-on-bone contact during joint movement. Therefore, defects of cartilage can result from natural wear and tear, or from traumatic events, such as injuries or sudden changes in direction during sports activities. Overtime, these cartilage defects which do not always produce immediate symptoms, could lead to severe clinical pathologies. The emergence of induced pluripotent stem cells (iPSCs) has revolutionized the field of regenerative medicine, providing a promising platform for generating various cell types for therapeutic applications. Thus, chondrocytes differentiated from iPSCs become a promising avenue for non-invasive clinical interventions for cartilage injuries and diseases. In this review, we aim to highlight the current strategies used for in vitro chondrogenic differentiation of iPSCs and to explore their multifaceted applications in disease modeling, drug screening, and personalized regenerative medicine. Achieving abundant functional iPSC-derived chondrocytes requires optimization of culture conditions, incorporating specific growth factors, and precise temporal control. Continual improvements in differentiation methods and integration of emerging genome editing, organoids, and 3D bioprinting technologies will enhance the translational applications of iPSC-derived chondrocytes. Finally, to unlock the benefits for patients suffering from cartilage diseases through iPSCs-derived technologies in chondrogenesis, automatic cell therapy manufacturing systems will not only reduce human intervention and ensure sterile processes within isolator-like platforms to minimize contamination risks, but also provide customized production processes with enhanced scalability and efficiency.

**Graphical abstract:**

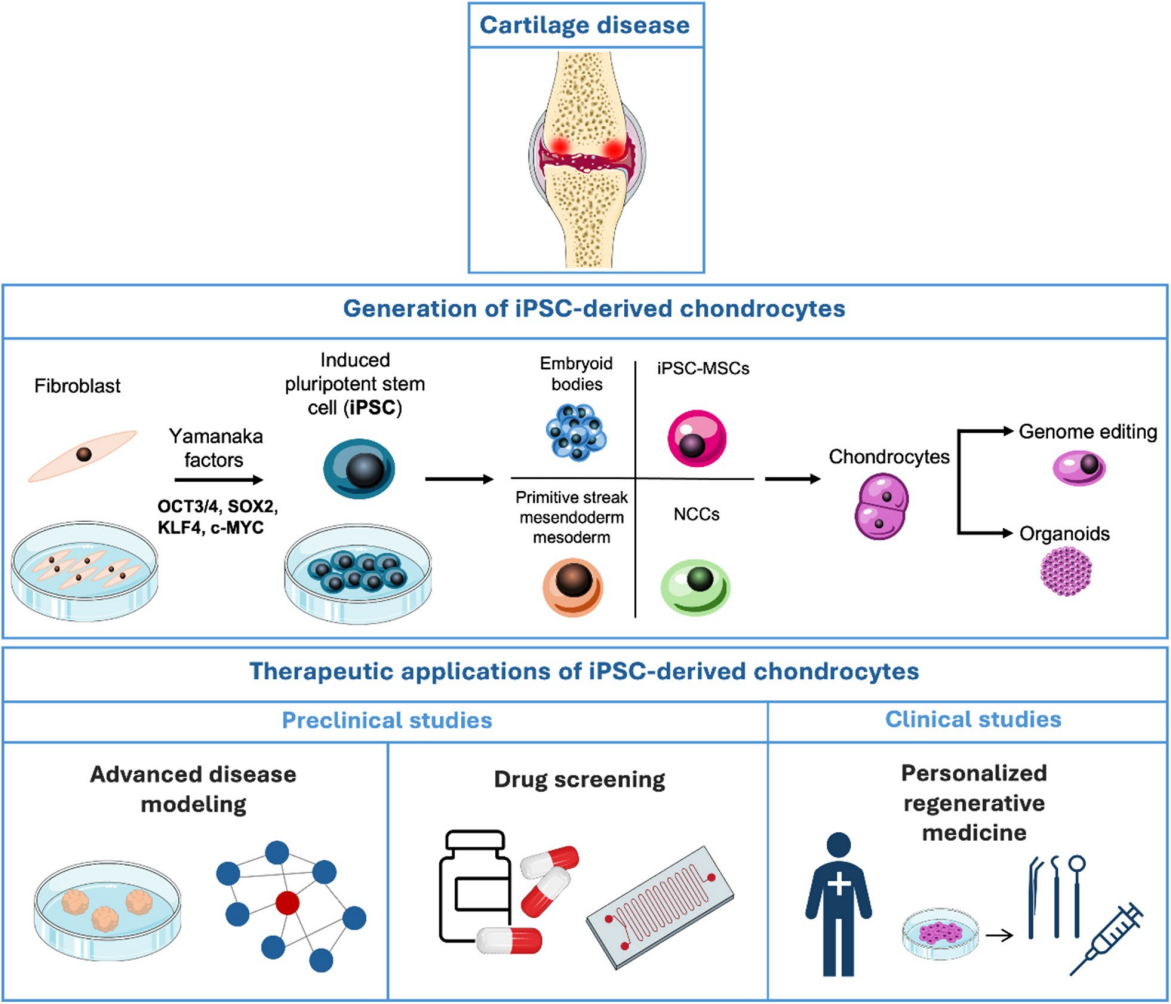

## Background

Cartilage is a semi-rigid, load-bearing, avascular connective tissue, formed solely by cells known as chondrocytes. These cells are loosely embedded in an extracellular matrix (ECM) composed predominantly of collagens and, in some cases, elastic fibers, hyaluronan and proteoglycans [1]. Cartilage formation, also known as chondrogenesis, is a dynamic cellular process of a condensed mesenchyme tissue derived from the mesoderm germ layer during embryogenesis. Cartilage represents the fetal precursor tissue for skeletal development. In adults, it persists at almost all joints between bones and in structures that must be deformable as well as strong such as in the respiratory system. Based on the structure and composition of their ECMs, chondrocytes form three different types of cartilage; namely, hyaline cartilage, fibrocartilage and elastic cartilage [2].

Cartilage exhibits diverse clinical aspects and relevance to various medical disciplines, including orthopedics, rheumatology, and respiratory medicine. Cartilage defects are associated with various clinical conditions such as osteoarthritis (OA), rheumatoid arthritis, and cartilage dysplasias [1]. Understanding the clinical significance of cartilage is critical for the development of effective therapeutics and interventions in various healthcare settings. Orthopedic surgeries such as joint arthroplasty and cartilage transplantation are the most commonly used therapeutic interventions for cartilage repair or replacement [3]. However, these surgical interventions are invasive or minimally invasive, and their ability to restore normal joint function, alleviate pain, and improve the quality of life for individuals with cartilage-related issues is limited.

Therefore, it is crucial to develop other non-invasive therapeutic approaches with high safety and efficacy. Theoretically and due to their ability to repair injured tissues, adult stem cells can be a good source for developing therapies for a large number of diseases [4]. Mesenchymal stem cells (MSCs) which can be derived from various tissues such as bone marrow, adipose tissu, placenta, umbilical cord blood, and multiple dental tissues, are multipotent cells that have the potential to differentiate into the mesenchymal lineages including osteocytes, chondrocytes, and adipocytes, as well as other non-mesenchymal lineages, such as cardiomyocytes, astrocytes, neural cells, and endothelial cells [5, 6]. Therefore, extensive efforts have been spent to develop MSCs-based cell therapies for a broad spectrum of diseases, encompassing cartilage and bone diseases, hematological diseases, inflammatory diseases, and graft-versus-host disease [7]. It is important to note that different transcription factors regulate the differentiation of MSCs to different lineages. Chondrogenic differentiation is determined by members the SOX (sex determining region Y (SRY)-related HMG-box) family of transcription factors SOX9, SOX5, and SOX6 while regulation of osteoblast differentiation involve the transcription factors runt-related transcription factor 2 (RUNX2), osterix, and β-catenin [8, 9]. Among the different sources of MSCs, bone marrow-derived MSCs (BM-MSCs) are the most commonly used MSCs in regenerative medicine, particularly for cartilage and bone regeneration [10]. Although significant strides have been taken to improve the chondrogenic differentiation from BM-MSCs and other cell sources, several obstacles persist complicating the achievement of consistent and effective chondrocytes required for clinical application [11]. Several factors may lead to the failure of utilizing BM-MSCs for efficient treatment of cartilage diseases including but not limited to the restricted proliferation capabilities in cultures [12], donor variations, and immunogenicity triggered during culture and cryopreservation [13].

These challenges could be addressed by the induced pluripotent stem cell (iPSC) technology. iPSCs are pluripoent cells which have the capacity for self-renewal and differentiation into almost all cell types [14]. The concept of self-renewal is the ability of the cells to undergo infinite cell divisions without differentiation into other cell types, while pluripotency is the ability of the cells to produce specialized cells of the three embryonic layers: ectoderm, mesoderm, and endoderm [15]. iPSCs can be generated from any type of cells through non-integrating reprogramming method using specific transcription factors known as Yamanaka factors namely, Octamer binding transcription factor 3/4 (OCT3/4), SOX2, Krüppel-like factor 4 (KLF4), and Cellular-Myelocytomatosis c-MYC [15]. Simplicity and reproducibility are the attractive features of the iPSC technology and have attracted the biomedical scientists to generate and differentiate iPSCs from numerous normal and disease-specific cell types for disease modeling and drug screening applications [16]. Syngeneic non-integrated iPSCs and their derivatives have no or minimal immunogenic effect supporting the notion that these cells could be used for cellular therapy without causing harmful immune responses [17]. Therefore, generation of iPSC-derived chondrocytes has become indispensable to advance our understanding of the mechanisms of cartilage-related disorders and represents an important avenue in regenerative medicine. In the following section, we will summarize different strategies developed to differentiate iPSCs into chondrocytes aiming to recapitulate the in vivo microenvironment that support chondrogenesis, and to generate functional and stable iPSC-derived chondrocytes.

## Main text

### Generation of iPSC-derived chondrocytes

**Fig. 1:**
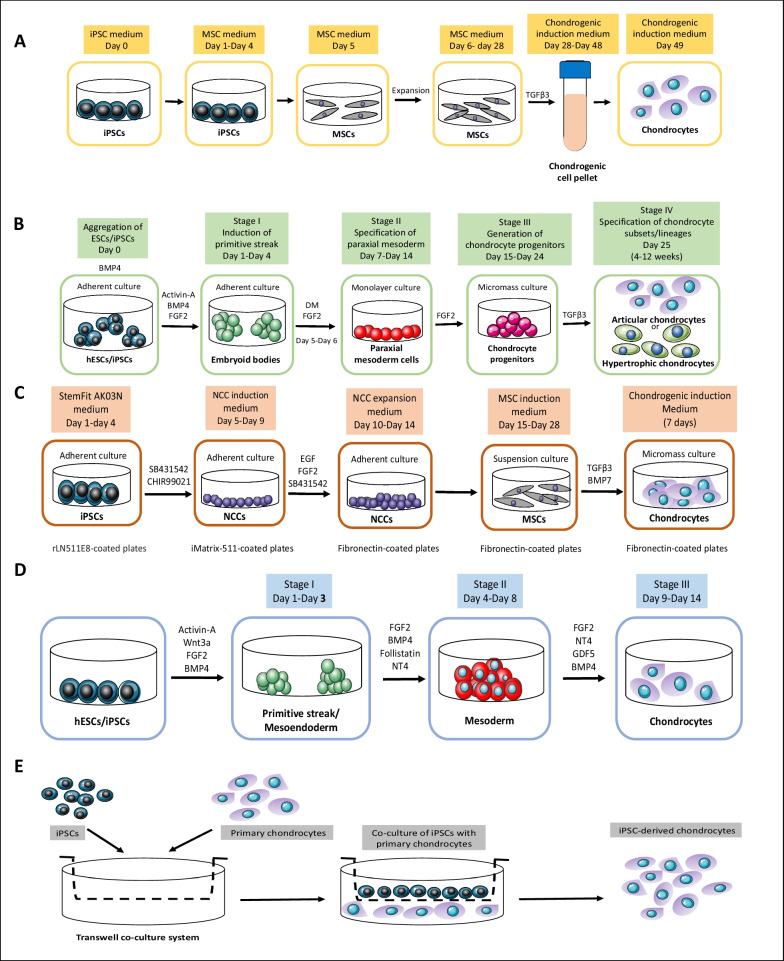
Schematic representation of the current strategies for in vitro differentiation of iPSCs to chondrocytes. **A** Via iPSC-derived MSCs. **B** Via EBs formation. **C** Via induction of NCCs. **D** Via primitive streak-mesendoderm and mesodermal lineage. **E** Via co-culture with primary chondrocytes. BMP4: bone morphogenetic protein 4; BMP7: bone morphogenetic protein 7; CHIR99021: glycogen synthase kinase 3 (GSK-3) inhibitor; DM: dorsomorphin; EB: embryoid body; EGF: epidermal growth factor; FGF2: fibroblast growth factor 2; GDF5: growth/differentiation factor-5; hESC: human embryonic stem cell; iPSC: induced pluripotent stem cell; MSC: mesenchymal stem cell; NCC: neural crest cell; NT4: neurotrophin-4; PDGF: platelet-derived growth factor; PSC: pluripotent stem cell; SB431542: transforming growth factor-beta receptor inhibitor; TGF-β3: transforming growth factor-beta 3; Wnt3a: Wingless/Int1 family member 3A

Chondrocytes can be differentiated from iPSCs though different intermediate stages, such as iPSC-derived MSCs (iPSC-MSCs), embryoid bodies (EBs) formation, induction of neural crest cells (NCCs), and primitive streak-mesendoderm and mesodermal lineage. iPSC-MSCs are morphologically highly similar to BM-MSCs and their gene expression profiling is also comparable to that of BM-MSCs [18], and exhibit traits that encompass features of both iPSCs and MSCs. iPSC-MSCs show reduced immunogenicity as compared to iPSCs [19], which renders them appropriate for allogeneic transplantation and enables development of off-the-shelf therapies. Moreover, patient-specific iPSC-MSCs open up the potential for developing personalized medicine for autologous transplantation, in vitro disease modeling, and drug screening [20]. These iPSC-MSCs were reported to differentiate into chondrocytes with growth factors, such as transforming growth factor-beta 3 (TGF-β3) (Fig. 1A). Another commonly used approach to obtain chondrocytes from iPSCs in vitro is through formation of three-dimensional (3D) aggregates of pluripotent stem cells (PSCs) known as embryoid bodies (EBs) (Fig. 1B). The EB has the capacity to generate ectodermal, mesodermal and endodermal cells due to its initiation of a process that resembles gastrulation-like events in embryonic development [21]. Several protocols have been developed under this category with slight variations in the number and concentration of growth factors used, the number of days required and whether an additional step such as differentiation of EBs to MSCs or paraxial mesoderm cells, is needed to differentiate iPSCs to chondrocytes [22]. NCCs are a multipotent group of transient embryonic cells in the vertebrate. They are derived from the ectoderm and differentiate to the peripheral nervous system cells and several non-neural cell types including pigment cells, and the cranio-facial cartilage and bones [23]. Taking the advantage of being multipotent, chondrogenic cells could be differentiated from the NCC-derived MSCs [24] (Fig. 1C). Chondrocytes were also reported to be differentiated from human embryonic stem cells (hESCs) through primitive streak or mesendoderm to mesoderm [25]. Cheng et al. followed this method to differentiate iPSCs to chondrocyte in three short stages using different combination of growth factors in each stage [26] (Fig. 1D). iPSCs can also be differentiated to chondrocytes by co-culture with primary chondrocytes (Fig. 1D). This method is based on the fact that the primary chondrocytes secret paracrine factors which may induce chondrogenic differentiation of the stem cells by closely mimicking the in vivo tissue microenvironment for chondrogenesis [27]. Moreover, co-culture permits crosstalk between the stem cells and the primary chondrocytes influencing chondrocyte development. It facilitates physical contact between different cell types which stabilizes the cellular phenotype and allows for communication of molecular signals involved in chondrogenic differentiation [28].

The above-mentioned studies showed that cartilage cells differentiated from human iPSCs represent a promising tool for regenerative medicine to treat cartilage-related diseases, however some challenges remain. The variability in the quality and characteristics of different iPSC lines affects the efficiency and consistency of chondrogenic differentiation [29]. Since the suspension culture promotes the chondrogenic differentiation and enables removal of non-chondrocytic cells, Yamashita and colleagues reported that homogenous chondrogenic nodules derived from iPSCs cultivated in suspension culture has the potential to form scaffold-free hyaline cartilage in animal models [30]. How to generate homogenous cartilage cells without formation of hypertrophic chondrocytes which have the potential to trigger the process of initiating endochondral ossification in vivo remains the main challenge. Moreover, iPSCs have the potential to form teratomas, therefore it is crucial to ensure complete elimination of undifferentiated iPSCs from chondrogenic cultures to prevent teratoma formation upon transplantation [31]. Obtaining fully mature chondrocytes from iPSCs with a phenotype comparable to native chondrocytes, is challenging [32]. In addition, undesired development of chondrogenic hypertrophy and fibrocartilage in vitro may require modification of the growth factors cocktail used [33]. Due to bovine xenoproteins, use of fetal bovine serum (FBS) in cell culture may induce adverse response in transplant patient upon injection of MSCs [34]. Additionally, there is a risk of infection because of viral and prion contamination [35]. Interestingly, MSC induction in xeno-free conditions may tackle these problems and promote the safety and efficiency of iPSC-MSCs for clinical applications [36].

### Genome-edited iPSC-derived chondrocytes

In the last decade, the clustered regularly interspaced short palindromic repeats (CRISPR-Cas9) approach has become an efficient and indispensable tool in biomedical research, and has been extensively explored in bone and cartilage research [37, 38]. It has been used to edit genes associated with chondrogenic differentiation to enhance their expression [39] or to modify signaling pathways involved in chondrogenesis [40]. For example, chondrogenesis can be regulated by the expression of SOX9 and Stat3 [39]. Chondrogenic differentiation of MSCs can be promoted by knocking down the *RUNX2*, a key transcription factor associated with osteoblast differentiation [41]. Genomic editing in iPSC-derived chondrocytes has been also reported in disease modeling. Efficient editing of cartilage related genes enables to investigate in depth the mechanisms underlying cartilage disorders and to identify potential therapeutic agents [42]. An interesting genome editing study showed simultaneous SOX9 activation and peroxisome proliferator-activated receptor gamma (PPAR-γ) repression in rat BM-MSCs, which promoted chondrocytes differentiation and regeneration of calvarial bone [43]. Various studies have investigated diverse targets for regeneration, paving the way for potential clinical trials in the near future. Genome editing has been employed to boost the regenerative potential of chondrocytes. This may involve editing genes related to ECM production, cell proliferation, or resistance to hypertrophy [41, 44, 45]. Although numerous studies have been reported on the application of genome-edited chondrocytes for in vivo cartilage repair, drug screening, and disease modeling [39, 41, 43], relatively few studies have been conducted specifically on iPSC-derived chondrocytes [40, 46, 47]. It was revealed that mutations in *TRPV4* disrupted the bone morphogenetic protein (BMP) signaling pathway in iPSC-derived chondrocytes and blocked formation of hypertrophic chondrocytes providing potential targets for drug development for TRPV4-associated skeletal dysplasias [48]. The existing methods for chondrogenic differentiation from iPSCs may generate heterogeneous cell populations. To resolve this problem, a collagen, type II, alpha 1- green fluorescent protein (COL2A1-GFP) knock-in reporter allele generated by CRISPR-Cas9 system was used to purify the cells. The purified chondroprogenitors exhibited enhanced chondrogenic potential in comparison to unselected groups [40].

Transplantation of allogeneic human iPSC-derived cartilage have shown to be more effective than allogeneic BM-MSC-derived cartilage [49]. However, these cartilage cells can trigger immunological reactions [50]. To overcome this issue, it is necessary to reduce the immunological reactions. The β2 microglobulin, a component of MHC class I molecules, was knocked down in monkey iPSCs before their differentiation into chondrocytes. As expected, the allogeneic iPSC-derived cartilage transplanted in osteochondral defects in monkey knee joints showed increased proliferation of natural killer cells and leukocytes surrounding the knocked down PSC-derived cartilage. This indicates the intricate processes in the immune response of the transplanted allogeneic cartilage in osteochondral defects in vivo [47]. These studies highlight the tremendous advantages of the CRISPR-Cas9 system in understanding the pathogenesis, identification of promising drug targets, and development of feasible treatment interventions for cartilage diseases.

### Cartilage organoids formed and differentiated from iPSCs

iPSC-derived cartilage organoids are 3D cell clusters that are created by differentiation of iPSCs in vitro. To support formation of cartilage organoids and their ability to self-renewal and self-organization, a number of biocompatible materials are used, such as Matrigel and synthetic hydrogels [51]. Cartilage organoid technology has been developed to facilitate drug screening through identification of important signaling pathways, recapitulate joint developmental events during embryogenesis and cartilage regeneration. Li and colleagues showed that long-term culturing of hiPSC-derived multi-tissue organoids (MTOs) in E8 medium results in a spontaneous emergence of hyaline cartilage tissues. Moreover, a transcriptome analysis indicated a strong association between the expression of chondrogenic markers in MTOs and fetal lower limb chondrocytes [52]. Another intriguing research demonstrated that subcutaneous implantation of iPSC-derived cartilage microtissues combined with pre-hypertrophic cartilage organoids in nude mice results in formation of both cartilaginous and bony regions [53]. Similarly, O’Connor and colleagues established osteochondral organoids using murine iPSCs through time-dependent sequential exposure of TGF-β3 and BMP2, to mimic natural bone development through the process of endochondral ossification. The generated organoids showed dual tissues consisting of cartilaginous and calcified bony regions [54]. A recent study showed a sequential differentiation process to produce matrix-rich cartilage spheroids from iPSC-MSCs by inducing NCCs in xeno-free environments. Efficient chondrogenic differentiation was induced by a thienoindazole derivative, TD-198946, a small molecule used to enhance differentiation of various human progenitor cells to chondrocytes. No hypertrophy, fibrotic cartilage formation, or dedifferentiation detected in vivo in the generated cartilage spheroids. These chondrogenic spheroids can serve as building blocks for biofabrication of engineered cartilage tissues, as they have the ability to fuse within a short timeframe of a few days [24]. It is worth mentioning that iPSC-derived cartilage organoids have also been reported to recruit osteogenic precursors for bone repair [55]. A recent study has revealed that allogeneic iPSC-derived cartilage organoids transplanted in the knee joints of a primate model of chondral defects integrated with articular cartilage of the host and prevented further degeneration of the surrounding cartilage [49]. These findings open new horizons for development of complex tissue engineered implants to promote zone-specific functionality by using pre-differentiated organoids as building blocks to establish articular cartilage grafts. Even though the research on iPSC-derived cartilage organoids is still in its infancy and creating fully functional cartilage organoids is still challenging, it is evident that they have demonstrated promising applications in drug screening, disease modeling, regeneration, and repair. It is of note that application of 3D bioprinting technology in development of iPSC-derived cartilage organoids can create more complex cartilage organoids and heighten their structural organization [56].

### Therapeutic applications of iPSC-derived chondrocytes

#### Advanced disease modeling

iPSC-derived chondrocytes have been utilized to recapitulate cartilage injuries and diseases in vitro (Table 1). The pluripotency and unlimited self-renewal capacity of the iPSCs make these cells vitally important for disease modeling, which permit us to investigate the mechanisms of various diseases, screen for potential treatment targets, and test therapeutic agents [57]. iPSC-derived disease models for both monogenic and complex cartilage diseases have been developed with more focus on single gene cartilage disorders [58]. Saitta et al. established an iPSC-based in vitro model of skeletal dysplasia to investigate the initial stages of abnormal cartilage formation. Mutations in the calcium channel gene *TRPV4* lead to abnormal chondrogenesis during cartilage growth plate differentiation [59]. Isogenic iPSCs with wild-type or mutant *NLRP3* have been generated from patients with neonatal-onset multisystem inflammatory disease. Both in vitro and in vivo chondrogenic differentiation were performed. Furthermore, immunodeficient mice that received mutant cartilaginous pellets in vivo experienced disordered endochondral ossification [60]. In vitro models of familial osteochondritis dissecans (FOCD) was developed using both patient BM-MSCs and iPSCs derived from patient fibroblasts to delineate the pathogenesis of this disease. The results showed that chondrogenic pellets with a high glycosaminoglycan (GAG) content but a poor structural integrity. Moreover, dysregulation of matrix production and assembly was evident. These findings show that how studying FOCD iPSC-derived chondrocytes can reveal insights into disease phenotype and pathogenesis offering a new in vitro model of OA and cartilage degeneration [61]. Esseltine et al. [62] converted fibroblasts from patient with oculodentodigital dysplasia (ODDD) into iPSCs, which provided a useful model for investigation of this disease. In this study, the iPSCs showed mutated *Cx43* gene, decreased levels of Cx43 mRNA and protein, resulting in impaired channel function. Furthermore, the subcellular localization of Cx43 changed during the chondrogenic differentiation of ODDD-derived iPSCs. This altered localization may have contributed to the more compact cartilage pellet morphology observed in differentiated ODDD-derived iPSCs. Additionally, other research teams successfully developed iPSC-derived disease models for other genetic and complex multifactorial skeletal disorders including type II collagenopathy*,* fibrodysplasia ossificans progressive (FOP), OA, hand OA, and early-onset finger OA (efOA) [58]. Recently, a novel method was introduced to direct iPSC-derived sclerotome through a sequential transformation in a 3D pellet culture. The generated chondroprogenitors can further be differentiated into articular chondrocytes or, alternatively, transformed into hypertrophic chondrocytes capable of transitioning into osteoblasts. Moreover, distinctive gene expression signatures have been identified at critical developmental stages, highlighting the effectiveness of this system in modeling genetic disorders affecting cartilage and bone [63]. In general, these studies demonstrated that normal chondrogenesis can be recapitulated using an iPSC-derived model, and disease-specific iPSCs exhibit molecular evidence of aberrant chondrogenic developmental processes. These findings may be utilized to develop therapeutic strategies for cartilage-related disorders.

**Table 1:** Cartilage-related preclinical studies using iPSC-MSCs and chondrocytes

	Condition(s)	Cell type(s)	Intermediate(s)	Chondrocyte cultivation	Application(s)	References	Year
iPSC-derived chondrocytes	Cartilage diseases	RVR *COL2A1*-GFP knock-in iPSC line and BJFF.6 iPSC line	Mesodermal cells	Pellet culture	Regenerative medicine	Dicks et al.[46]	2020
					Disease modeling		
	Hand osteoarthritis (HOA)	hiPSCs derived from patient’s dermal fibroblasts	EBs	Micromass culture	Disease modeling	Castro-Viñuelas et al.[104]	2020
	Osteochondral defects	CBMC-derived iPSC lines	EBs	Pellet culture	Regenerative medicine	Rim et al.[105]	2020
	Osteoarthritis (OA)	iPSC line derived from NHEKs	MSCs	Micromass culture	Regenerative medicine	Chang et al.[72]	2020
	Achondroplasia (ACH)	hiPSCs derived from patient’s dermal fibroblasts	Mesodermal cells	Suspension culture	Drug discovery	Ozaki et al.[106]	2020
	Early-onset finger osteoarthritis (efOA)	hiPSCs derived from patient’s dermal fibroblasts	EBs	Pellet culture	Disease modeling	Rim et al.[107]	2021
	Multiple epiphyseal dysplasia (MED) and metaphyseal chondrodysplasia type Schmid (MCDS)	iPSCs derived from patients’ dermal fibroblasts and PBMCs	Mesodermal cells	Suspension culture	Disease modeling	Pretemer et al.[108]	2021
					Drug discovery		
	Achondroplasia (ACH)	hiPSCs derived from dermal fibroblasts	EBs	Suspension culture	Drug discovery	Kimura et al.[109]	2021
	Chondoral defect	hiPSC line (414C2)	NCCs, MSCs	Pellet culture	Regenerative medicine	Nakamura et al.[64]	2021
	Osteochondral defect	CBMC-derived iPSC lines	EBs	Pellet culture	Regenerative medicine	Lee et al.[110]	2021
	Osteochondral defect	Mouse gingiva-derived iPSCs	–	Pellet culture	Modeling skeletal development	Zhang et al.[65]	2022
					Disease modeling		
					Drug discovery		
	Genetic cartilage and bone disorders	hiPSC lines	Paraxial mesoderm-derived sclerotome	Pellet culture	Modeling skeletal development	Lamandé et al.[63]	2023
					Disease modeling		
					Drug discovery		
	Articular cartilage defect	*Cynomolgus monkey* iPSC line (1466A1)	Mesodermal cells	Suspension culture	Regenerative medicine	Abe et al.[49]	2023
iPSC-derived MSCs	Intervertebral disc degeneration (IVDD)	hiPSs	MSCs	–	Regenerative medicine	Sun et al.[79]	2021
	Cartilage defects	hiPSCs	NCCs, MSCs	Spheroid culture	Regenerative medicine	Zujur et al.[24]	2023

2D: two dimentional; 3D: three dimentional; ACH: achondroplasia; BM-MSCs: bone marrow-derived mesenchymal stem cells; CBMC: umbilical cord blood mononuclear cell; COL2A1: collagen, type II, alpha 1; CS: chitosan; EBs: embryoid bodies; efOA: early-onset finger osteoarthritis; FOP: fibrodysplasia ossficans progressive; GFP: green fluorescent protein; hESCs: human embryonic stem cells; hiPSCs: human induced pluripotent stem cells; HOA: hand osteoarthritis; IVDD: intervertebral disc degeneration; MCDS: metaphyseal chondrodysplasia type Schmid; MED: multiple epiphyseal dysplasia; MSCs: mesenchymal stem cells; NCC: neural crest cell; NHEK: Normal human epidermal keratinocytes; OA: osteoarthritis; PBMC: peripheral blood mononuclear cell; TD: thanatophoric dysplasia

To overcome some limitations of scaffold-based 3D cell culture method, scaffold-free methods showed promising results as well. Nakumora et al. [64] reported efficient fabrication of unified, self-sufficient, and functional cartilaginous constructs by combining iPSCs and bio-3D printers using a Kenzan needle array technology. This approach may facilitate repairing of articular cartilage defects*.* Zhang et al. [65] established a rapid and efficient approach, employing a 3D rotary suspension culture system, to directly guide iPSC differentiation toward the chondrogenic mesoderm lineage. Subsequently, the research group introduced a tetracycline-controlled *BMP4* gene regulation system for iPSCs, linking transcriptional activation of BMP4 with heightened chondrogenesis using the piggyBac (PB) transposon-based gene delivery system. Kotaka and associates used magnetically-labeled iPSCs and an external magnetic force to evaluate the safety and efficacy of magnetic field-mediated delivery of iPSCs for articular cartilage repair in nude rats. The results demonstrated the effectiveness and safety of this approach for in vivo cartilage repair [66]*.*

#### Drug screening

Surgical interventions are performed to prevent progressing of focal articular cartilage defects [29], however, no effective drugs are available for treatment of cartilage regeneration. Using human MSCs for screening of compounds that promote chondrogenesis has limitations due to limited expansion of MSC passages, variations between donors and the high cost [67]. The development of the iPSC technology and advancement in genome editing approaches provide crucial tools for drug screening by establishing iPSC-derived chondrocytes. Using human iPSCs, a 96-well screening platform was developed to identify chondrogenesis-inducing agents that can be used separately or combined with other techniques for cartilage regeneration and repair. Due to their ability to promote chondrogenesis in vitro and in vivo, AB235 and NB61, two chimeric ligands of Activin/BMP2, were used and tested separately at two different doses for validation of the 96-well chondrogenic screening format. Strikingly, elevated concentrations of each of these two agents resulted in improved chondrogenic differentiation [68]. Another OA drug screening study was conducted on iPSC-derived or native mouse cartilage samples. The inflammatory environment of OA was induced in these cells by interleukin-1α (IL-1α), and a 96-well plate format was used for screening of OA drug candidates. The high-throughput screening revealed that the nuclear factor kappa-light-chain-enhancer of activated B cells (NF-κB) inhibitor SC514 was the most effective drug candidate to reduce cartilage loss induced by IL-1α [69]. Increased mineralization in the FOP-derived iPSCs has been detected, a phenomenon that could be mitigated by the use of the BMP inhibitor DMH1 [70]. It has been demonstrated that statins could effectively rectify the degraded cartilage observed in both chondrogenically differentiated thanatophoric dysplasia type 1 (TD1)- and achondroplasia (ACH)-specific iPSCs [71]. These studies illustrate the potential of iPSCs to provide a suitable platform to identify novel therapeutic agents for cartilage-related disorders and facilitate development of personalized regenerative medicine.

### Preclinical studies

Chondrocytes derived from iPSCs have demonstrated great promise in a variety of regenerative medicine applications, especially in relation to cartilage regeneration and repair [49, 64, 72]. These cells offer regenerative treatments for diseases such as OA and cartilage injuries (Table 1). They can be combined with biomaterial scaffolds or scaffold-free methods to create engineered cartilage grafts for transplantation [73]. Generation of cartilage tissues from patient-specific iPSCs reduces the risk of immunological rejection, thus this personalized strategy has a potential for treating diseases such as OA [19]. Before their clinical application, preclinical studies of the iPSC-derived chondrocytes are crucial to assess their viability, functionality, and safety [74]. iPSC-MSCs were used to repair cartilage defects in a rabbit model. Macroscopic and histological assessment revealed more cartilage repair in the experimental group as compared to both the control and scaffold implantation group. Furthermore, no teratoma formation detected in all the three groups indicating the safety and potential of iPSC-MSCs for cartilage regeneration [75]. Ko et al. [76] implanted iPSC-derived chondrocytes in osteochondral defects in immunosuppressed rats. The defects exhibited a significantly higher quality of cartilage repair than in the control. In another study, homogenous cartilaginous particles derived from chondrocyte-specific reporter hiPSC lines were transplanted into joint surface defects in immunodeficient rat and immunosuppressed mini-pig models. The neocartilage survived and integrated into native cartilage, and no tumor formation was observed in all the animal models following the transplantation [30]. The potential of MSC-based therapies is attributed to the release of trophic factors via paracrine signaling, with small extracellular vesicles (sEVs) potentially playing a significant role [77]. Zhu et al. [78] investigated the therapeutic efficacy of exosomes derived from synovial membrane MSCs (SM-MSC-Exos) and iPSC-MSCs (iPSC-MSC-Exos) in treatment of OA. The injected exosomes in an OA mouse model showed that iPSC-MSC-Exos exhibit a stronger therapeutic impact on OA compared to SM-MSC-Exos. Similarly, iPSC-MSC-derived sEVs injected in degenerative discs of intervertebral disc degeneration (IVDD) rat models revealed significant improvement in IVDD and senescence of nucleus pulposus cells of the IVD [79]. Given the poliferative capacity of autologous iPSC-MSCs, these cells ensure a consistent and abundant source of therapeutic sEVs, which could introduce a new therapeutic strategy for OA and IVDD treatment [78, 79]. As previousely mentioned, Nejadnik et al. developed an effective method to directly differentiate human iPSCs (hiPSCs) into MSCs and chondrocytes without the need for EBs formation. Transplantation of these cells in OA rat models successfully repaired the osteochondral defects [33]. However, the traces of fibrocartilage and hypertrophic cartilage detected in the generated chondrocytes in vitro and use of FBS in the chondrogenic medium may prevent their clinical application. Use of Xeno-free media and thorough characterization of hiPSC-derived MSCs and chondrocytes will be essential prior to transplantation [33]. An intriguing study has demonestrated that chondrogenic spheroids derived from iPSC-MSCs retain cartilage phenotype in vivo comparable to the chondrogenic-like tissues generated from the same cell spheroids in vitro. In contrast to spheroids obtained from iPSC-MSCs, distinct bone-like tissue formation was evident in BM-MSC spheroids. This may prove the capacity of iPSC-MSC-derived chondrogenic spheroids to form cartilage-like tissues without endochondral ossification for treatment of cartilage defects in vivo [24]. Additionally, due to the ability of chondrogenic spheroids to fuse rapidly within a short timeframe, they can serve as as building blocks for constructing larger cartilage tissues using techniques like the Kenzan bioprinting method [56]. Current focus tends to shift towards investigating immune reactions in the context of allogeneic cartilage transplantation. Abe and colleagues were the first to conduct allogeneic cartilage transplantation into a primate model using major histocompatibility complex (MHC)-mismatched iPSC-derived cartilage organoids without the need for immunosuppressive drugs [49]. Remarkably, the transplanted organoids exhibited successful engraftment into chondral defects on the knee joint surface of the primate model, demonstrating survival, integration, and remodeling similar to native cartilage, without any observed immune reactions [49]. The findings of these preclinical studies demonstrate effective and clinically translatable approaches for regenerating cartilage tissue using hiPSC-derived MSCs and chondrocytes, offering potential enhancements in cartilage regeneration outcomes in cartilage diseases.

### Clinical studies

Over the past decade, iPSCs have shown significant advancements, offering new prospects for personalized cell therapy. Patient-derived iPSCs exhibit a lower risk of rejection compared to allogeneic iPSCs. Therefore, some challenges such as tumorigenicity or immunogenicity must be addressed before the iPSCs can be extensively utilized in clinical therapy. To date, 89 clinical trials referenced under “induced pluripotent stem cells” have been registered on the World Health Organization (WHO)-managed main databases (https://clinicaltrials.gov/, International Clinical Trials Registry Platform (ICTRP), https://trialsearch.who.int/). Several studies from the Japan Primary Registries Network (https://rctportal.niph.go.jp/en) can be added to the list since most of their 21 iPSCs trials are not cross-referenced with the WHO’s platforms. Among the total 110 identified clinical trials, 51 trials were registered as interventional and the remaining as observational. Despite the low rejection risk, slow shifting from autologous to allogenic iPSC-derived therapy approach has been crucial due to the time and cost required for characterization and safety testing of each cell line. Furthermore, allogeneic iPSCs approach allow more time for the testing process, and once an approved cell line is established, it can be used to treat multiple patients. Opting for allogeneic cell therapy would result in a readily accessible therapeutic product for interventions [80].

Until recently, pluripotent cell-derived MSCs were not a popular focus in clinical research, with only a small number of studies exploring this area, despite the wide variety of potential tissues that could be produced. Currently, only three clinical trials involving ESC-derived MSCs [81–83], and six iPSC-MSCs clinical trials have been reported (Table 2) [84, 85]. It is important to note that from the six clinical trials, cartilage regeneration through iPSC-MSCs was only addressed in two studies. In 2020, the University of Sydney and Cynata Therapeutics conducted phase 1 clinical trial to evaluate the safety, efficacy, and cost-effectiveness of an allogenic MSCs therapy (Cymerus MSCs) for tibiofemoral knee OA [86]. Lately, Cynata Therapeutics has reported that 321 subjects were recruited for the phase 3 SCUlpTOR clinical trial which will start in 2024 for 24 months (Trial ID: ACTRN12620000870954). In the foreseeable future, the phase 1 clinical trial sponsored by the Chinese Nuwacell Biotechnology company will investigate the safety and efficacy of the NCR100 allogenic iPSC-MSCs intra-articular injection for treatment of knee OA (Trial ID: NCT06049342). This is the first Chinese iPSC-derived cell product approved to be used in phase 1 clinical trial following six years of research and development, (https://en.nuwacell.com/news). It is to be noted that a study tried to directly differentiate allogenic iPSCs into chondrocytes without intermediate MSCs differentiation, to treat knee OA as well (Trial ID: jRCTa050190104). The 2020 Japanese interventional trial from Kyoto University was followed by a second observational trial in 2020 for post-treatment evaluation on the subject’s knees (Trial ID: jRCT1050220051).

**Table 2:** Cartilage-related clinical trials using iPSC-MSCs and chondrocytes

	Trial ID	Title	Recruitment Status	Estimated number of participants	Phase	Cell type	Study type	Condition	Start date (estimated)	Country	Sponsor
iPSC-derived MSCs	NCT06049342	A phase I, open label, single arm, multiple center, dose escalation clinical trial to evaluate the safety, tolerability and efficacy of human iPSC-derived MSCs (NCR100) injection in the treatment of subjects With KOA	Not yet recruiting	12	Phase 1	Allogenic	Interventional	KOA	25.01.2024	China	Nuwacell Biotechnologies Co., Ltd
	https://clinicaltrials.gov/										
	ACTRN12620000870954	Evaluating the efficacy and cost-effectiveness of stem cell injections in people with mild to moderate knee osteoarthritis: a randomised placebo-controlled trial (The SCUlpTOR trial)	Recruiting	440	Phase 3	Allogenic	Interventional	KOA	15.03.2021	Australia	Cynata Therapeutics Limited
	https://anzctr.org.au/ACTRN12620000870954.aspx										
iPSC-derived chondrocytes	JPRN-jRCTa050190104NIPH^a^	A clinical study for treatment of articular cartilage damage in knee joints with allogeneic iPSC-derived cartilage. -TACK-iPS	Terminated	4	N/A	Allogeneic	Interventional	KOA	11.11.2020	Japan	Matsuda Shuichi, Kyoto University Hospital
	https://trialsearch.who.int/Trial2.aspx?TrialID=JPRN-jRCTa050190104										
	JPRN-jRCT1050220051	Evaluation of knee function and safety after treatment of articular cartilage damage in knee joints with allogeneic iPSC-derived cartilage	Recruiting	4	N/A	Allogenic	Observational	KOA	16.06.2022	Japan	Matsuda Shuichi, Kyoto University Hospital
	https://jrct.niph.go.jp/en-latest-detail/jRCT1050220051										

ID: identification number; iPS: induced pluripotent stem; iPSC: induced pluripotent stem cell; KOA: Knee osteoarthritis, N/A: not applicable

^a^This clinical trial was discontinued

As a concluding remark, there have been no results regarding cartilage regeneration through iPSC-derived cell therapy in these trials so far. The scarcity of iPSC-MSCs and cartilage-oriented clinical trials indicates significant potential for further advancement and enhancement. Hopefully with the extensively growing iPSCs research, cartilage regeneration for condition such as OA will receive greater attention.

### Limitations of iPSC-derived chondrocyte in vitro* models*

Throughout this review, numerous studies have demonstrated the tremendous advantages offered by iPSC-derived chondrocytes for cartilage research. However, there are some limitations associated with iPSC-derived chondrocyte in vitro models. The first limitation is that the iPSC-derived chondrocytes may show an immature phenotype, and it is still challenging to obtain iPSC-derived chondrocytes with full maturation and stability [87]. The second limitation is the possibility to generate diverse cell populations with variation in maturation stages. This heterogeneity might complicate result interpretation and compromise the validity and reproducibility of experimental results [22]. Due to the potential of iPSCs to form teratomas, residual undifferentiated iPSCs in iPSC-derived cartilage grafts may pose a risk of tumor formation in transplantation studies [88]. Another main challenge is the variability in the efficiency of chondrogenic differentiation among different iPSC lines and even among clones of the same line [31]. Moreover, the culture conditions for differentiation of iPSCs to chondrocytes may not fully replicate the complex microenvironment of native cartilage tissue. The artificial culture conditions can influence cellular behavior and might not fully capture the in vivo physiological and mechanical complexity of chondrocytes [18, 24]. Even though patient-derived iPSCs can potentially reduce the immunological rejection [89], the in vitro differentiation and manipulation processes may introduce foreign antigens, raising concerns about the immunogenicity of the generated chondrocytes [19]. In addition, the ability of iPSC-derived chondrocytes to produce a mature and robust ECM may be limited. The structure and organization of the ECM are essential for the functionality and integrity of cartilage tissue. Therefore, ECM defects may affect the utility of in vitro models [90]. Last, but not the least, the robustness of cartilage in vitro models may be affected by the technical aspects of iPSC maintenance, differentiation, and characterization, which may introduce variability [32]. These limitations illuminate the challenges associated with iPSC-derived chondrocyte in vitro models. Improvement and optimization of chondrogenic differentiation protocols may overcome these limitations and ensure reliable and comparable results across various studies.

### Scaling-up of iPSC-derived cells

The potential of iPSC-derived technologies in chondrogenesis, offering significant benefits for OA and other medical conditions, is evident. However, unlocking these benefits encounters hurdles such as limited process understanding, outdated manufacturing techniques, and insufficient automation. Manual manufacturing and quality control processes prove labor-intensive and error prone. To address the anticipated demand for iPSC-derived cells, scalable production methods must be developed to uphold clinical-grade yields and immunomodulatory properties. Moreover, research indicates that human iPSCs might present an epigenetic edge compared to adult stem cells in producing chondrocytes on a large scale without a tendency towards hypertrophy. Ko and his team showcased heightened expression of key chondrogenic markers such as SOX9, COL2A1, and aggrecan (ACAN), alongside decreased levels of hypertrophic markers like COL10A1 and RUNX2 in iPSC-derived chondrocytes when compared to BM-MSC pellets [76].

It is crucial to establish robust protocols for large-scale iPSC production to support tasks like cell banking. Thorough evaluations of iPSC-derived chondrocytes in large-scale production settings are essential for consistent quality outcomes and to tackle the challenge of spontaneous differentiation. Closing the gap between research and clinical application necessitates the development of scaled production technologies spanning from initial seeding to final fill-and-finish stages. Embracing full automation in iPSCs cell therapy manufacturing and quality control is paramount for enhancing both product quality and production efficiency in this rapidly evolving field [91]. A recent study developed hiPSC-derived limb bud mesenchymal cells (ExpLBM cells) with strong chondrogenic potential and stable proliferation. Using a stirred bioreactor, this method outperformed conventional culture plate methods by yielding significant cartilage tissue with just 1 × 10^6^ cells. This produced significant amounts of cartilaginous particles, suggesting a scalable method for cartilage regeneration without immune rejection. This efficient approach requires minimal cell quantities and offers potential scalability through adjustments in medium volume and cell numbers [92]. Another recent study has introduced GelMA microcarriers developed via step emulsification microfluidic devices as a degradable platform for amplifying iPSC-MSCs in scalable bioreactors, while maintaining typical MSC traits and immune-modulatory capabilities. These GelMA microcarriers, manufactured with efficiency and reproducibility in mind, facilitate substantial expansion of iPSC-MSCs (up to 16 times within 8 days) in vertical wheel bioreactors, with a post-digestion viability exceeding 95%. When compared to monolayer culture, iPSC-MSCs expanded on GelMA microcarriers exhibit at least similar, if not superior, immune-modulatory potential. This approach marks a notable progression in producing immune-modulatory iPSC-MSCs, providing scalability, cost-efficiency, and simplified cell retrieval through direct dissolution of microcarriers, thereby minimizing cell wastage [93].

A novel, good manufacturing practice (GMP)-compliant scalable manufacturing procedure is introduced for the fabrication of iPSC-MSCs, tackling the aforementioned hurdles. By employing xenogeneic-, serum-, and feeder-free conditions, alongside chemically defined maintenance for iPSCs, the process eliminates the necessity for murine feeders and accomplishes mesoderm induction, resulting in heightened performance of MSCs in immunopotency assessments. The manufacturing process comprises three phases: iPSC banking, iPSC expansion and differentiation into MSCs, and MSC expansion and formulation of the final clinical product. Impressively, one vial of iPSCs can yield an average of 3.2 × 10^10^ MSCs, and the complete iPSC bank has the potential to generate 2.9 × 10^15^ MSCs, equating to 29 million clinical doses, each containing 1 × 10^8^ MSCs. This method presents a promising resolution to the challenges of supply, scalability, and consistency in iPSC-MSC production, paving the way for their utilization in clinical applications with heightened efficacy and safety. This optimized manufacturing process for iPSC-MSCs has been applied in treating steroid-resistant acute graft versus host disease (SR-aGvHD) in a phase 1 clinical trial but could be similarly employed in the iPSC-MSCs-Chondrocyte approach for chondrogenesis [84].

The aim of automating cell therapy manufacturing is to reduce human intervention, ensuring sterile processes within isolator-like platforms to minimize contamination risks. Despite notable advancements, challenges persist, including difficulties in executing specific biological procedures with robotic assistance, prompting the need for exploring new solutions and standardization. Establishing an automated manufacturing platform requires precise definition of process parameters and configurations through validated standard operating procedures (SOPs). To address these needs, an advanced automated cell manufacturing platform was employed to produce both equine and human iPSC-MSCs via EBs [94]. These iPSC-MSCs were further demonstrated their ability to differentiate into adipogenic, osteogenic, and chondrogenic lineages proficiently. The main goal of this study was to develop a simplified and uniform procedure for isolating MSCs from peripheral blood under GMP conditions, ensuring their viability and purity. Compared to existing protocols documented in the literature, this approach offers simplicity, scalability and consistently delivering robust cell purity [94]. Recently, another automatic system was reported to produce iPSC-derived therapies, covering a range of cell types including iPSC-MSCs, iPSC-derived chondrocytes, and extracellular vesicles [95]. iPSC expansion and differentiation into MSCs and chondrocytes take place in plates, while expansion of iPSC-derived MSCs and production of extracellular vesicles utilize microcarriers within stirred tank bioreactors. The system is designed to oversee iPSC expansion, differentiation, and the fill and finish of the products. Furthermore, this platform including a range of quality control assays such as microscopy, cell counting, viability assessment, qPCR, and endotoxin assays, aims to address these challenges by establishing an automated platform for producing cell therapies specifically targeting OA, and serves as an example of how existing automation technology can be customized and improved to enhance scalability and efficiency.

## Conclusions

Genomic abnormalities detected during the reprogramming and subsequent expansion of iPSCs raised serious safety concerns [96]. Therefore, several factors including starting cell source, method of delivery, reprogramming factor and cell passage, should be taken into consideration for the generation of iPSCs in order to reduce not only genomic instability [97], but also immunogenicity [98, 99].

The field of iPSC-derived cartilages is rapidly evolving, and several approaches and perspectives have been explored to tackle limitations and enhance the potential applications of these cells in regenerative medicine. Development of new or optimization of the current differentiation protocols to improve the maturation and stability of iPSC-derived chondrocytes is critical [25]. This can be achieved by further research on signaling pathways, culture conditions, and other factors that facilitate the maturation of iPSC-derived chondrocytes. It is significantly important to implement cutting-edge 3D culture systems combined with ink-free bioprinting technique to more closely mimic the in vivo microenvironment of cartilage tissue [56]. Using bioreactors, biomimetic scaffolds, 3D bioprinting and other advanced technologies can improve the functional characteristics of iPSC-derived chondrocytes for cartilage repair. Generation of heterogeneous cell populations remains one of the major challenges in development of efficient cartilage grafts [100]. To eliminate undesired cells and promote the homogeneity of iPSC-derived chondrocyte populations, sustained development of precise genome editing tools is quite essential. Moreover, it is necessary to identify the sources of heterogeneity in iPSC-derived chondrocyte populations to reduce variability and improve reproducibility [101]. Tumorigenicity associated with residual undifferentiated iPSCs can be addressed by advancements in purification methods and genetic modifications to increase the safety of iPSC-derived chondrocytes for clinical applications [102]. Moreover, scalability and cost-effectiveness of the methods used for generation of iPSC-derived chondrocytes should be improved by simplifying the differentiation protocols, optimizing culture conditions, and utilizing automation technologies [95]. Additionally, it is very crucial to enhance the development of in vivo models to investigate the safety and efficacy of iPSC-derived chondrocytes in preclinical studies [103]. Successful preclinical studies should be followed by well-designed clinical trials in patients with cartilage-related disorders. Furthermore, for personalized regenerative medicine, the design of preclinical and clinical trials should focus on the integration of patient-specific iPSCs with advanced gene editing technologies and highly efficient chondrogenic differentiation protocols. These future perspectives reflect the continuous endeavors to harness the full potential of iPSC-derived chondrocytes, opening the door for innovative approaches in cartilage regeneration and repair. Since this field is advancing rapidly, interdisciplinary collaborations and advancement in technologies will play a vital role in shaping the future of iPSC-based cartilage regeneration research.

## Abbreviations

2D: Two dimentional
3D: Three dimentional
ACH: Achondroplasia
BM-MSCs: Bone marrow-derived Mesenchymal stem cells
BMP2: Bone morphogenetic protein 2
BMP4: Bone morphogenetic protein 4
CBMC: Umbilical cord blood mononuclear cell
c-MYC: Cellular-Myelocytomatosis
COL2A1: Collagen, type II, alpha 1
COL2A1-GFP: Collagen, type II, alpha 1-green fluorescent protein
CRISPR: Clustered regularly interspaced short palindromic repeats
CS: Chitosan
DMH1: Dorsomorphin homolog 1
EBs: Embryoid bodies
ECM: Extracellular matrix
efOA: Early-onset finger osteoarthritis
FOP: Fibrodysplasia ossficans progressive
GMP: Good manufacturing practice
hESCs: Human embryonic stem cells
hiPSCs: Human induced pluripotent stem cells
HOA: Hand osteoarthritis
ID: Identification number
iPSCs: Induced pluripotent stem cells
IVDD: Intervertebral disc degeneration
KLF4: Krüppel-like factor 4
KOA: Knee osteoarthritis
MCDS: Metaphyseal chondrodysplasia type Schmid
MED: Multiple epiphyseal dysplasia
MSCs: Mesenchymal stem cell
N/A: Not applicable
NCCs: Neural crest cells
NF-κB: Kappa-light-chain-enhancer of activated B cells
NHEKs: Normal human epidermal keratinocytes
OA: Osteoarthritis
OCT3/4: Octamer binding transcription factor 3/4
PBMCs: Peripheral blood mononuclear cells
PSCs: Pluripotent stem cells
RUNX2: Runt-related transcription factor 2
sEVs: Small extracellular vesicles
SOPs: Standard operating procedures
SOX: SRY-related high mobility group box
SRY: Sex determining region Y
TD1: Thanatophoric dysplasia type 1
TGF-β3: Transforming growth factor-beta 3
